# Synthesis and characterization of novel denosumab/magnesium-based metal organic frameworks nanocomposite prepared by ultrasonic route as drug delivery system for the treatment of osteoporosis

**DOI:** 10.3389/fbioe.2023.1153969

**Published:** 2023-05-30

**Authors:** Fahad Alsaikhan, Mustafa Z. Mahmoud, Muath Suliman

**Affiliations:** ^1^ College of Pharmacy, Prince Sattam Bin Abdulaziz University, Alkharj, Saudi Arabia; ^2^ Department of Radiology and Medical Imaging, College of Applied Medical Sciences in Al-Kharj, Prince Sattam bin Abdulaziz University, Al-Kharj, Saudi Arabia; ^3^ Department of Clinical Laboratory Sciences, College of Applied Medical Sciences, King Khalid University, Abha, Saudi Arabia

**Keywords:** bone, denosumab, drug delivery system, metal organic framework, osteoporotic

## Abstract

**Introduction:** The metal-organic frameworks (MOF) have shown fascinating possibilities in biomedical applications, and designing a drug delivery system (DDS) based on the MOF is important. This work aimed at developing a suitable DDS based on Denosumab-loaded Metal Organic Framework/Magnesium (DSB@MOF (Mg)) for attenuating osteoarthritis.

**Materials and Methods:** The MOF (Mg) (Mg3(BPT)2(H2O)4) was synthesized using a sonochemical protocol. The efficiency of MOF (Mg) as a DDS was evaluated by loading and releasing DSB as a drug. In addition, the performance of MOF (Mg) was evaluated by releasing Mg ions for bone formation. The MOF (Mg) and DSB@MOF (Mg) cytotoxicity towards the MG63 cells were explored by MTT assay.

**Results:** MOF (Mg) characterized by using XRD, SEM, EDX, TGA, and BET. Drug loading, and releasing experiments proved that DSB was loaded on the MOF (Mg) and approximately 72% DSB was released from it after 8 h. The characterization techniques showed that MOF (Mg) was successfully synthesized with good crystal structure and thermal stability. The result of BET showed that MOF (Mg) had high surface areas and pore volume. This is the reason why its 25.73% DSB was loaded in the subsequent drug-loading experiment. Drug release and ion release experiments indicated DSB@MOF (Mg) had a good controlled release of DSB and Mg ions in solution. Cytotoxicity assay confirmed that the optimum dose of it had excellent biocompatibility and could stimulate the proliferation of MG63 cells as time went on.

**Conclusion:** Due to the high loading amount of DSB and releasing time, DSB@MOF (Mg) can be promising as a suitable candidate for relieving bone pain caused by osteoporosis, with ossification-reinforcing functions.

## 1 Introduction

Denosumab, or DSB, as a human monoclonal antibody can target the RANKL (Receptor Activator of Nuclear Factor-kappa B ligand) that has a role in the differentiation, performance and survival of osteoclasts (Sugimoto, 2011; Dupont et al., 2022). RANKL, is a protein normally expressed on the surface of stromal cells and osteoblasts, and mediates osteoclast differentiation and osteolytic bone resorption (Kong et al., 1999; Wang, et al., 2022a). Denosumab is indicated for postmenopausal women with osteoporosis at high risk of fracture, or for patients who have failed or are intolerant to other available osteoporosis therapies. Osteoporosis Canada Clinical Practice Guidelines identify denosumab as a first-line option for preventing vertebral, hip and non-vertebral fractures (Hanley et al., 2012). It is a common anti-resorptive agent to manage osteoporosis and skeletal-related events (such as pathological fractures) in people suffering from bone metastases or lesions (such as multiple myeloma) (Sanchez-Rodriguez et al., 2020; Terpos et al., 2021; Xu et al., 2021; Zheng et al., 2022; Li et al., 2023). In February 2009, the United States Food and Drug Administration (FDA) accepted Amgen’s Biologic License Application (BLA) for denosumab for the treatment of PMO and bone loss due to hormone ablation therapy for prostate and breast cancer (Pageau, 2009). DSB binds to RANKL and thus prevents bone resorption induced by osteoclast, which enhances Bone Mineral Density (BMD) and suppresses Bone Turnover Markers (BTM). The DSB dosage is determined on the basis of the appeared indication, for example, 60 mg every 6 months subcutaneously to treat osteoporosis (Cummings et al., 2009; Yulin Li et al., 2022). People with osteoporosis are mostly elderly, and thus long-term oral intake of DSB may be associated with gastrointestinal-related clinical complications (Ma et al., 2015; Zhang et al., 2020; Zhou S. et al., 2021; Wang, et al., 2022b). Accordingly, there is a need to develop a safe approach to deliver DSB in the treatment of bone pain induced by osteoporosis. Osteoporotic pain during osteoporosis can be caused by inflammation and bone loss, which can also affect inflammation and bone resorption and aggravate the severity of osteoporosis (Redlich and Smolen, 2012; Loi et al., 2016; Chesi et al., 2019; Wang et al., 2020; Cao et al., 2022; Xu et al., 2022). Hence, blocking inflammation and alleviating bone loss may be involved in reducing bone pain and treating osteoporosis.

Researchers in crystal engineering and material chemistry have recently focused on metal-organic frameworks (MOFs) due to their unique structure and attractive applications in catalysis, drug delivery, separation and gas storage (Li et al., 1999; Banerjee et al., 2008; Hinks et al., 2010; Yang et al., 2010; Zhang et al., 2022; Cheng et al., 2023). Reportedly, multiple MOFs can act as DDSs, and exert specific therapeutic impacts owing to the metal ions released by the framework disintegration (Li et al., 2020; Chen et al., 2022).

Magnesium (Mg) is one of the key elements required for the normal production of bone matrix (Zhao et al., 2017; Zhou W. et al., 2021; Lei et al., 2022), which is involved in osteoblast adhesion, proliferation and growth, and extra mineralization of bone (Venkatraman and Swamiappan, 2020), and suppression of inflammation through down-regulating pro-inflammatory indices and up-regulating anti-inflammatory cytokines (Sun et al., 2020). Thus, the MOF (Mg) is expected to act as an admirable carrier for Mg and DSB delivery.

Accordingly, the hypotheses of this research are: a) MOF (Mg) can be a potent carrier for safe delivery of DSB; b) Magnesium in MOF (Mg) can exert positive biological activities in osteoporosis such as anti-inflammatory responses and bone formation; and c) DSB-loaded MOF (Mg) can be a stable DDS to comprehensively manage osteoporosis. For this purpose, an attempt was made to make MOF(Mg) loaded with DSB named DSB@MOF(Mg). Diverse approaches were employed to systematically determine the chemical and physical profiles of DSB@MOF(Mg), and the MTT method was applied to investigate its biosafety.

## 2 Materials and methods

### 2.1 Devices and chemicals

All materials were of analytical grade with no need for further purification. Biphenyl-3,4′,5-tricarboxylate (>96.0%), Magnesium nitrate (=99.999%), N,N-dimethylformamide (>99.8%), Methyl thiazolyl tetrazolium (MTT) (>98.0%), fetal bovine serum (FBS) (>90.0%), alpha-minimum essential medium (α-MEM) and penicillin-streptomycin belonged to Sigma–Aldrich Company (Germany). Dimethyl sulfoxide (>99% DMSO) and Phosphate-buffered saline (premixed powder × 1 PBS) belonged to Sangong Co., Ltd (China).

We explored the product organization by recording X-ray diffraction (XRD) pattern using Philips analytical PC-APD X-ray diffractometer with graphite mono-chromatic Cu (α1, λ1 = 1.54056 Å) and Kα (α2, λ2 = 1.54439 Å) radiation. We also observed the MOF (Mg) using the scanning electron microscopy (SEM) and energy dispersive X-ray spectroscopy (EDX) (KYKY & EM 3200). We applied a STA-1500 thermoanalyzer to perform thermal behavior analysis in N2 between room temperature and 350°C. We determined the content of DSB@MOF (Mg)-released Mg in PBS using Inductively coupled plasma mass spectrometry (ICP-MS; Agilent; 7700 series; the United States). We detected the amounts of DSB loading and release using high-performance liquid chromatography (HPLC; Agilent 1260 LC; the United States). MTT assay (International Standard Organization; ISO 10993-5:2009 protocol) used for testing the biotoxicity of MOF(Mg) and DSB@MOF(Mg).

### 2.2 Preparing the MOF (Mg)

We synthesized the MOF (Mg) (Mg3(BPT)2(H2O)4) according to a sonochemical protocol of biphenyl-3,4′,5-tricarboxylate with magnesium nitrate in 5:1 N,N-dimethylformamide (DMF)/H2O under 20-min ultra-sonication at irradiation power of 450 W (Guo et al., 2009). The product was colorless block crystals with a yield of 81%.

### 2.3 Producing the DSB@MOF (Mg) composite

To fabricate the as-proposed DDS, DSB (3 g) was poured into ethanol (20 mL) and stirred at ambient temperature with 600 rpm. After complete dissolution of DSB, 300 mg of MOF (Mg) was appended while continuously stirring at ambient temperature with 600 rpm for 24 h. The solution was centrifuged and the supernatant was harvested. The composite particles were rinsed with deionized water for three times, and subsequently dried at 120°C for 24 h to reach the final product DSB@MOF (Mg).

### 2.4 Loading the drug

To measure the amount of DSB loading, 10 mg of DSB@MOF (Mg) was poured into 0.1 M NaOH (10 mL), stirred at ambient temperature for 30 min and centrifuged. Then, 1 mL of supernatant was dissolve in methanol (1 mL) and subsequently filtered via (a 0.45-µm membrane), followed by analyzing through HPLC. Thus, the specifications were Sunfire-C18 reverse-phase column (5 μm, 4.6 × 150 mm Waters), the mobile phase of V (acetonitrile): V (K2HPO4, pH = 2) = 1:1, the flow rate of 1 mL/min, the temperature of 25 °C, and the solution optical density (OD) of 260 nm using Ultraviolet–visible spectroscopy (UV–Vis). The concentration curve of standard DSB solution was applied to obtain the amount of DSB loading, based on the equation as follows (Li et al., 2019a):

$$DLE\%=\frac{M_{t}}{M_{s}}\times 100\%$$

Herein, Mt stands for total mass of DSB loaded, Ms for total MOF (Mg) content and DLE express Encapsulation/Entrapment Efficiency.

### 2.5 DSB release

A beaker containing PBS (50 mL) was added with 100 mg of DSB@MOF (Mg), followed by stirring constantly at 37°C with 300 rpm for 72 h. The supernatant (2 mL) was collected at each interval, and then the fresh PBS (2 mL) was poured into the beaker for keeping the solution equilibrium. The HPLC was utilized to determine the amount of DSB released from DSB@MOF (Mg) in PBS. The DSB release percentage (CR%) was computed, as follows (Li et al., 2019b):

$$CR\%=\frac{M_{r}}{W_{t}}\times 100\%$$

Herein, Mr stands for the DSB released mass and Wt for total mass of DSB loaded.

### 2.6 Release of Mg ions

A centrifuge tube (50-mL) with PBS (30 mL) was added with 100 mg of DSB@MOF (Mg), followed by continuously shaking with 100 rpm at 37°C for 72 h. The, the resultant product was centrifuged, and 3 mL of supernatant was discarded and replaced with 3 mL of fresh PBS for keeping the solution equilibrium. The supernatant filtering was performed by a 0.45-µm membrane, followed by detecting the Mg ion concentration via inductively coupled plasma mass spectrometry (ICP-MS) (Li et al., 2019a).

### 2.7 *In vitro* test for determining the cytotoxicity

The MOF cytotoxicity towards the MG63 cells was explored by MTT assay. Thus, MG63 cells (1 × 104 cells/well) were transferred into a 96-well plate and exposed to α-MEM with 10% FBS, 1% penicillin-streptomycin and 5% CO_2_ at 37°C for 24 h. Then, the cell medium was renewed with a fresh medium bearing variable MOF concentrations and co-cultured for one, three and 5 days. Next, it was added with 10 µL of MTT (5 mg/mL) and cultured for another 4 h. At last, the media was discarded and 100 µL of DMSO was appended for resolving the violet crystallization. The OD of all wells were detected at 490 nm by a microplate reader (iMark, Bio-Rad). All samples were explored in triplicate. Statistical analysis was carried out using Student’s t-test in R environment (V3.5.3), and a *p*-value less than 0.05 was considered to be statistically significant.

## 3 Results

**FIGURE 1:**
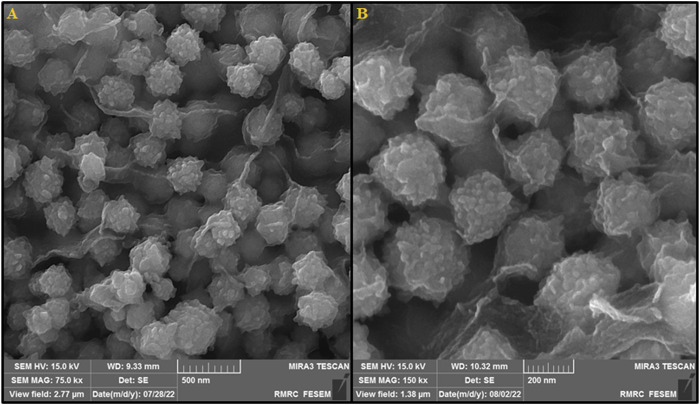
**(A)** SEM image showing the surface morphology of the MOF (Mg) and **(B)** high-resolution SEM image.

FE-SEM images displayed the morphology and microstructure of the as-developed MOF (Mg) (Figure 1). Figure 1 illustrates the microsphere-like morphology of MOF (Mg) with the mean diameter of about 250 nm. The MOF (Mg) microspheres had the rough surface carrying a mass of fine nanoparticles (NPs) (20-40 nm), with raspberry-like morphology.

**FIGURE 2:**
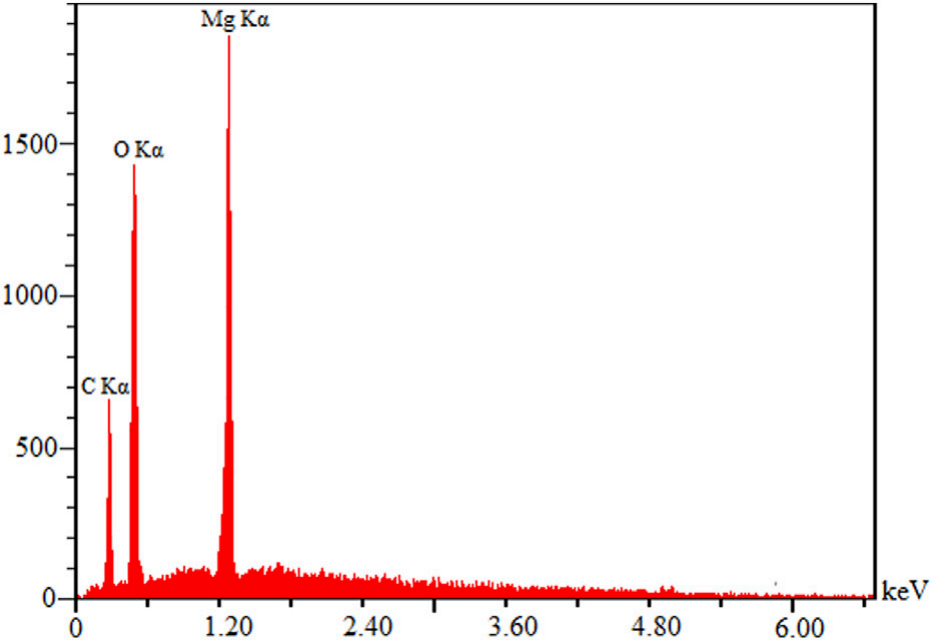
EDX spectra of MOF (Mg).

Figure 2 shows EDX data on the chemical analysis of MOF (Mg), the strong signal of which can be seen at 1.2-keV energy for Mg and the weak signals of which relate to C and O. The main emission energy at 1.2 keV meant the correct identification of Mg.

**FIGURE 3:**
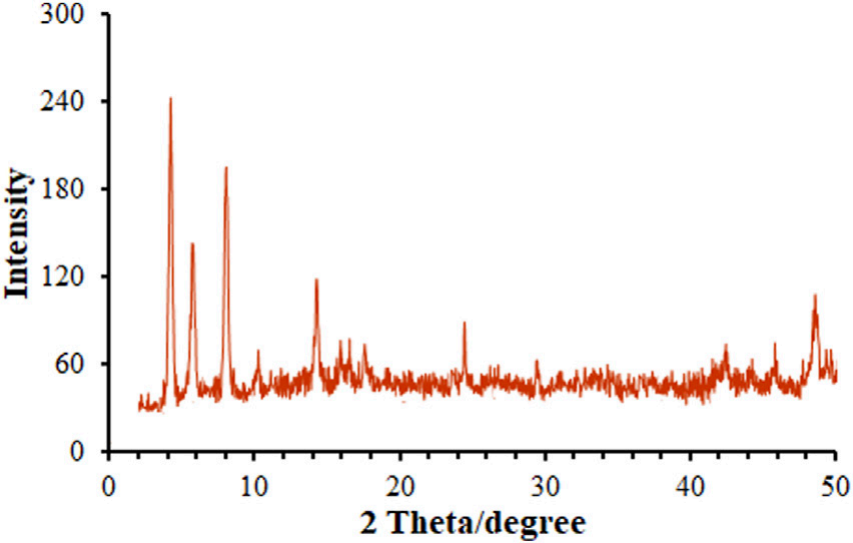
XRD pattern of MOF (Mg).

Figure 3 illustrates the XRD patterns captured for annealed samples, exhibiting several diffraction peaks related to MOF (Mg) as a poly-crystalline structure (Guo et al., 2009). The crystallite size was computed on the basis of Scherrer’s equation of D = Kλ/βcosθ; herein, K constant is 0.9, λ value is 1.54 Å, β stands for Full Width at Half Maximum (FWHM) obtained in radians and θ for Bragg’s diffraction angle. Hence, the MOF (Mg) size D) could be estimated simply. The mean crystallite size of MOF (Mg) was calculated to be 28.2 nm, meaning its commendable dispersity and crystal structure.

**FIGURE 4:**
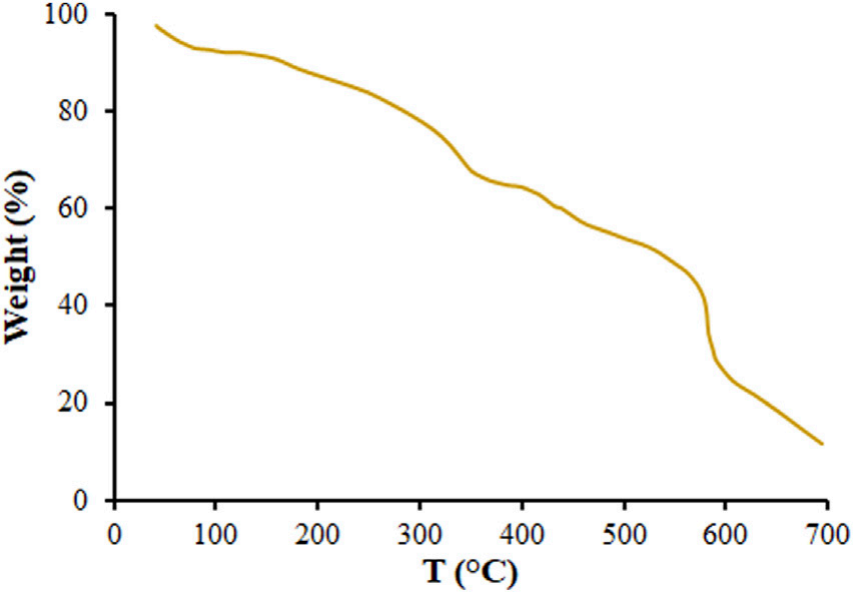
The TG analysis of the MOF (Mg).

Figure 4 presents the results of Thermogravimetric (TGA) analysis of MOF (Mg) to explore the thermal stability of the MOF (Mg). According to the DSC curve, the MOF (Mg) lost the incorporated water, which resulted in starting a complex endothermic decomposition at 50°C–90°C. A second weight loss can be seen for the MOF (Mg) after heating at 350°C assigned to lost biphenyl-3,4′,5-tricarboxylate (Guo et al., 2009; Geng et al., 2015; Geng et al., 2016).

**FIGURE 5:**
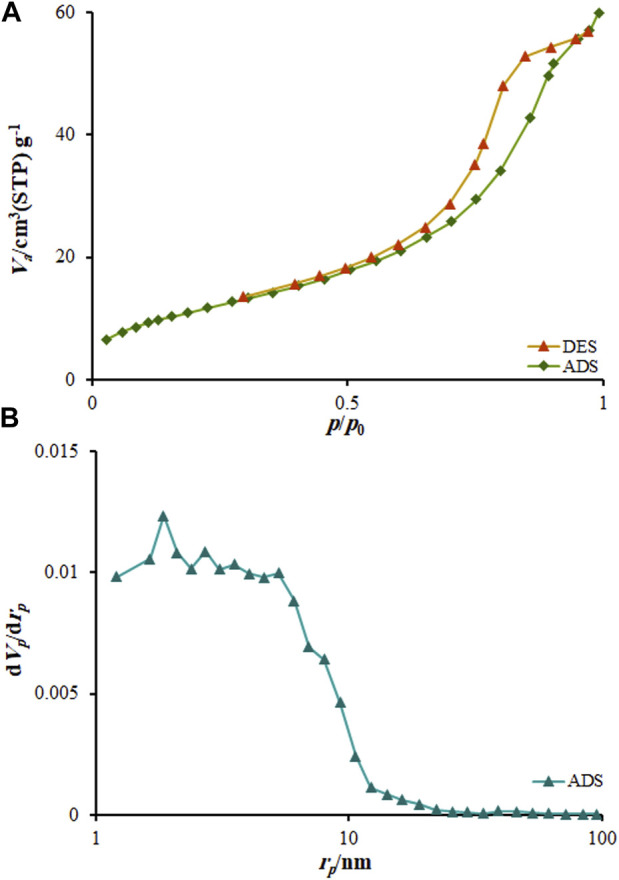
BET plot **(A)** N2 adsorption-desorption isotherms (P/P0, relative pressure) at 77 K MOF (Mg); **(B)** BJH results obtained for MOF (Mg).

The nitrogen isothermal adsorption–desorption determinations were applied to explore the porous nature of MOF (Mg). Figure 5A illustrates a typical IV isotherm having a clear hysteresis loop ranged from 0.31 to 1.0 pp0-1, which confirms the mesoporous architecture of nanocomposite. According to the distribution of pore size determined by desorption isotherm based on Barret–Joyner–Halenda (BJH) approach (Figure 5B), the as-produced MOF (Mg) exhibits a thin pore-size distribution centered at about 8.82 nm, further verifying the mesoporous architecture of nanocomposite.

**FIGURE 6:**
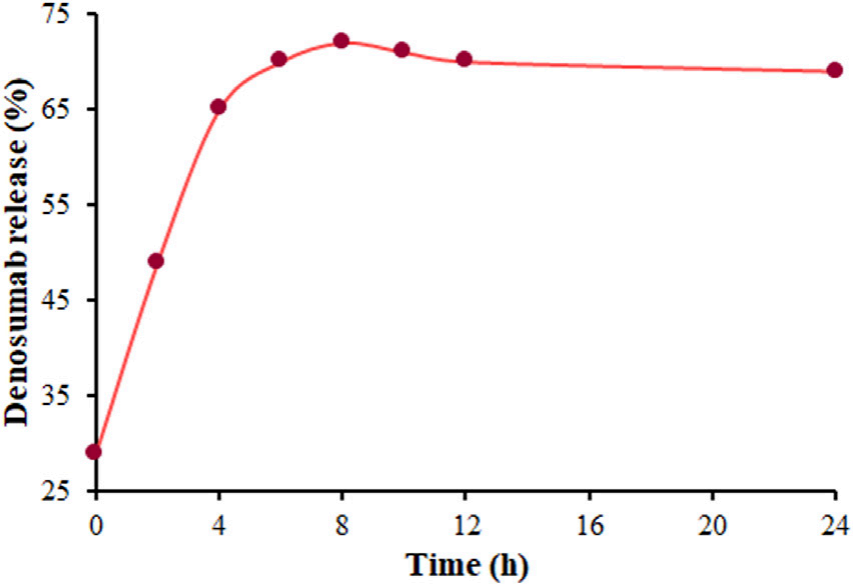
The release profile of DSB from DSB@MOF (Mg) in PBS.

The highest drug load is a pivotal factor in a DDS. Hence, the HPLC was utilized to explore the highest loading rate (DLE) of DSB on the MOF (Mg), which was obtained to be 25.73%. The HPLC was employed to explore the process of DSB release from DSB@MOF (Mg) in PBS. Figure 6 shows two-stage DSB release, including rapid release during the first 8 hours following the stop-release thereafter. The DSB release was about 72% within 8 hours, and stopped at around 24 h.

**FIGURE 7:**
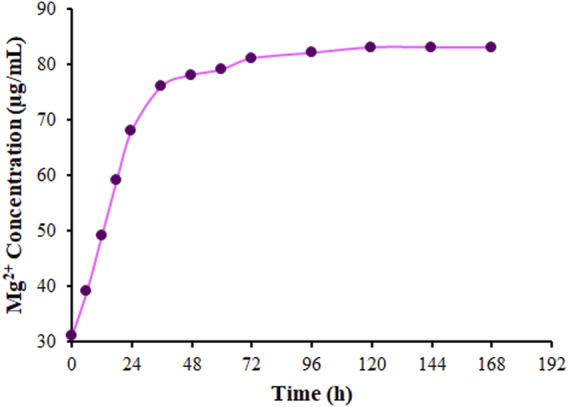
The release curve of Mg ions from DSB@MOF (Mg) in PBS.

The ICP-MS was applied to explore the Mg release from DSB@MOF (Mg) in PBS based on a certain time (Figure 7). The Mg releases quickly in the first 24 h and then slowly with time. The Mg content stabilizes gradually in the solution 72 h later. The DSB@MOF (Mg) could release Mg, which meant the potential possibility of accelerating bone formation.

**FIGURE 8:**
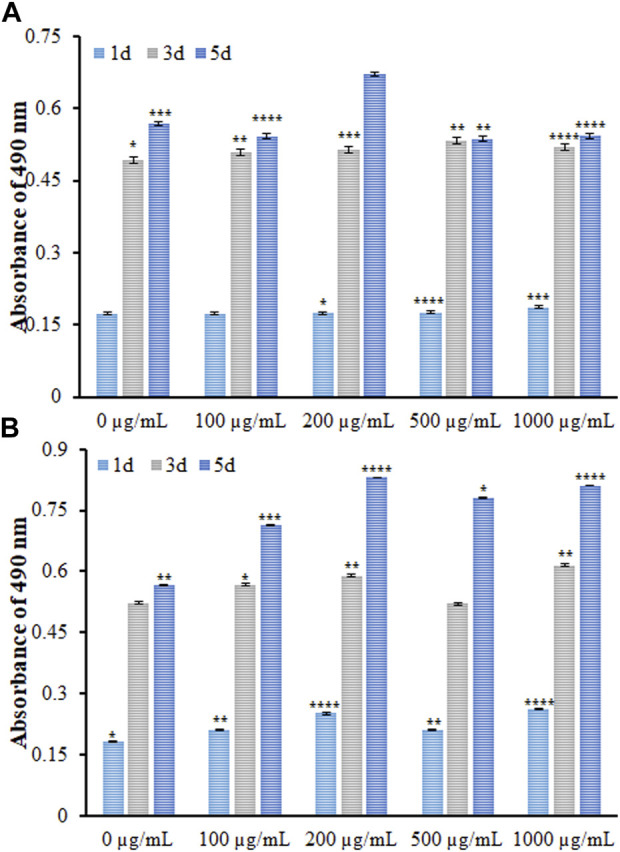
The viability of MG63 cells incubated with different concentrations of the **(A)** MOF (Mg) and **(B)** DSB@MOF (Mg) at different time intervals evaluated by MTT at the absorbance wave of 490 nm (* denotes *p*-value < 0.05, ** stands for *p*-value < 0.01, *** presents *p*-value < 0.001, **** means *p*-value < 0.0001).

The biocompatibility of DSB@MOF (Mg) was characterized by using MTT assay after the MG63 cells were treated with various concentrations of it for 1, 3, and 5 days (Figure 8).

## 4 Discussion

Given the characteristics and properties of MOFs and Mg ions, MOF (Mg) was prepared as a potential drug carrier. SEM, XRD, TGA and BET results proved that MOF (Mg) was successfully synthesized. The main idea of this work is to explore the capacity of MOF (Mg) as DSB carriers. HPLC experiment indicated that about 25.73% DSB were loaded on MOF (Mg). In addition, ICP-MS identified that DSB@MOF (Mg) continuously released Mg2+ for 72 h. Moreover, MTT was performed to identify the biocompatibility of MOF (Mg) and DSB@MOF (Mg), and to ensure them could be used as promising therapeutic agents for osteoporosis.

MOFs as a DDSs have been extensively studied for its high specific surface area, large porosity and adjustable chemical functions (Li et al., 2019b; Zhang et al., 2021). Due to special coordination features, they are easy to self-assemble into different shapes, such as cauliflower, needle, fusiform, bullet shell, etc. (Bao et al., 2011). While having a rough surface for a nanocarriers is not recommended; since it could cause inflammatory reaction when placed adjacent to body tissues. But researchers have found that if nanocarriers have high thermal and chemical stability, they do not cause inflammatory reactions (Patelli et al., 2018). For example, Ge et al. (2021) introduced a drug nanocarrier with a cauliflower-like morphology with high surface roughness for the treatment of osteoporotic pain. Sun et al. (2017) reported Cu-MOFs with combination structure of triangles and neglect the spherical structure in the center and their application as the transport vehicles for the delivery of Ibuprofen and doxorubicin hydrochloride. The TGA analysis showed that, MOF (Mg) had high thermal stability, which were all suitable for drug delivery at body temperature. Therefore, according to the above-mentioned and TGA analysis, it can be concluded that the rough surface of DSB@MOF (Mg) does not cause inflammatory reactions.

**FIGURE 9:**
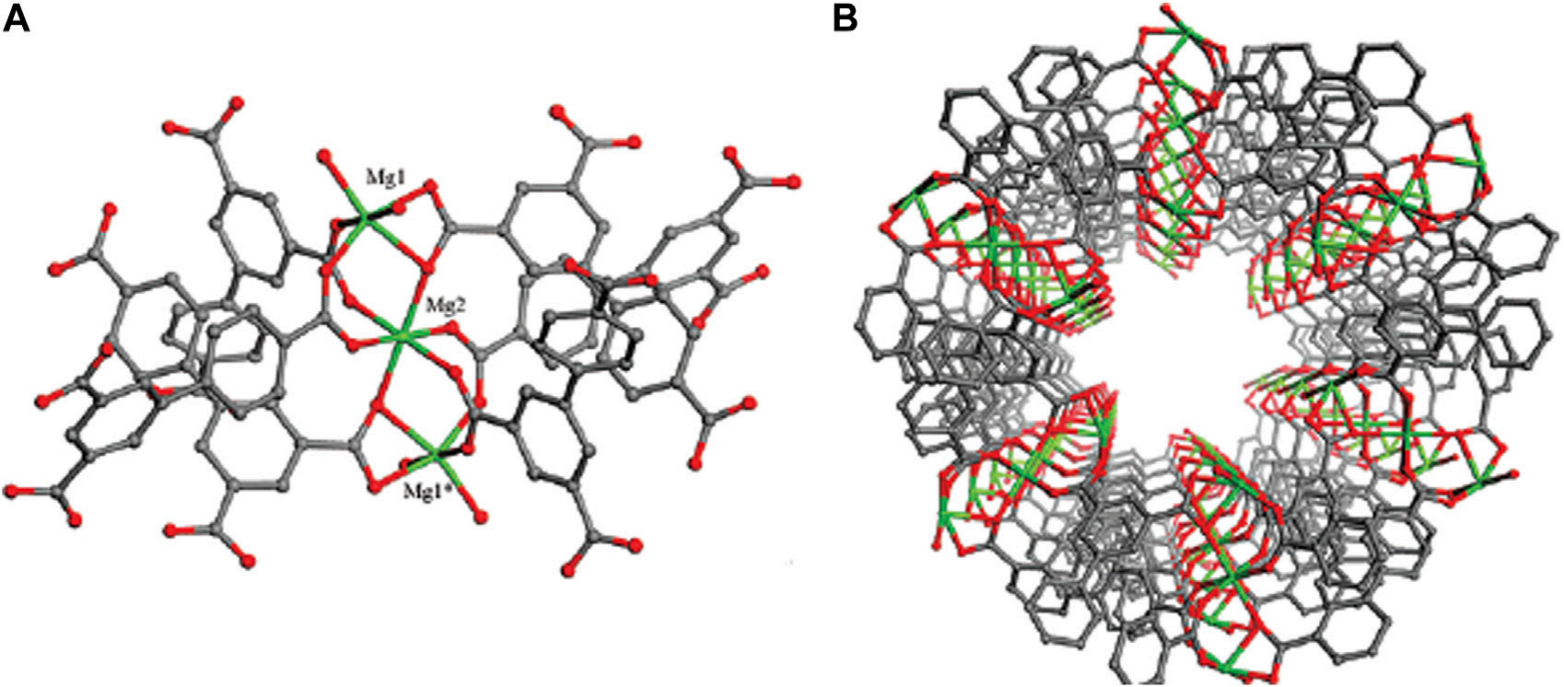
**(A)** Trinuclear magnesium carboxylate clusters and a representation of the inorganic six-coordinate SBUs in Mg^3^(BPT)2(H2O)4. **(B)** Network of Mg3(BPT)2(H_2_O)4 with a 1D hexagonal nanotube-like channel (Guo et al., 2009).

The Brunauer–Emmett–Teller (BET) specific surface area was computed to be 72.513 m2g-1 and pore volume was calculated to be 0.11 cm3g-1 for nanocomposite. The mesoporous formation had an association with the gas release within the precursor decomposition. BET surface of MOF (Mg) is much higher than other drug carriers (Ge et al., 2021; Hassanpouraghdam et al., 2022; Pooresmaili et al., 2023). Therefore, MOF (Mg) has high BET surface, which made them efficient for drug delivery. These results are confirmed by the 3D structure of MOF (Mg) shown in Figure 9. Mg(II) ions and organic linker in the MOF (Mg) structure are bound to chains and arranged in a parallel and hexagonal (Figure 9), one-dimensional pore, leading to large surface area and thus providing commendable adsorption capacity.

Reportedly, the drug release procedure can occur for three reasons of a) physical adsorption on the MOF surface, b) the interaction of drug molecules with MOF wall (such as hydrogen bonds) in the pore, and c) the carrier’s pore size. A rapid release rate can be seen for the physically adsorbed drug molecules on the surface and in the middle of the pores. The slower release rate can be seen for the drug on the pore walls due to the impacts of hydrogen bonding and pore adsorption (Li et al., 2019a). In this research, the fast release within the first 8 hours was due to the departure of the MOF pore-encapsulated DSB. The slow release in the second phase (between 8 and 24 h) was due to the release of DSB adsorbed on the wall with hydrogen bond breaking and MOF collapse. Since the release of DSB stops after 24 h, its long-term release was not investigated. Moreover, it is difficult to realize drug-release lasting for several days, due to the most of drugs exist on the surface and pore of MOFs. Even if long-term sustained drug-release is achieved, drug damage cannot be avoided (Ge et al., 2021).

Inorganic ions are considered as valuable therapeutic biomedical agents since they areperforming as enzyme co-factors and persuading the signalling reaction pathways and metabolites during hard tissue regeneration (Venkatraman and Swamiappan, 2020). The elements like calcium, magnesium and silicon play a preferred role in bone formation. Magnesium is required for the regulation of bone growth and repair (Padmanabhan et al., 2013). Therefore, DSB@MOF (Mg) should be a good DDS which can be release Mg for bone growth and repair.

In cytotoxicity assay no significant elevation or reduction was found in the proliferation of MG63 cells exposed to variable MOF (Mg) concentrations on days one and three. On the day 5, an insignificant reduction was found at various concentrations, except for 200 μg/mL, meaning a significant elevation. Concerning the DSB@MOF (Mg) group, slight elevation occurred in the MG63 cell proliferation with raising DSB@MOF (Mg) concentration on the day 1, and a significant elevation was found on the day 3. However, the MG63 cells in all levels had an amplification on the day 5. Data showed no cytotoxicity for DSB@MOF (Mg) and MOF (Mg). In addition, MTT assay was employed to evaluate and compare the biocompatibility of MOF (Mg) and DSB@MOF (Mg). By comparing the amount of viable cells of the samples at different times, it is observed that the biocompatibility of the MOF (Mg) (200 μg/mL) sample is close to 91.2% after 120 h incubation. The cloaking of DSB further enhanced the cytocompatibility of 200 μg/mL of MOF (Mg) produced a cell viability of 97.4% after 120 h incubation. The coating of DSB may pretend the MOF (Mg) as homologous substances, and endow the MOF (Mg) with biomimetic characteristics to improve their biocompatibility. According to ISO 10993-Part, if the cell viability is higher than 70% compared to the control sample, it can be said that the MOF (Mg) and DSB@MOF (Mg) are non-toxic and biocompatible. Cytotoxicity assay confirmed that the optimum dose (200 μg/mL) of it had excellent biocompatibility and could stimulate proliferation of MG63 cells as time went on.

## 5 Limitation and future prospects

In recent years, the use of MOFs as DDSs for on-demand drug release has gained increasing attention around the world. MOFs, demonstrate great potential in overcoming the limitations and drawbacks of conventional DDS for controllable spatiotemporal drug release to achieve good therapeutic efficacy. Although rapid progress has been made on the study of MOF-DDS, there are still many issues that should be addressed before their clinical application.

1) More studies should be focused on the preparation of MOF-DDS with low-toxicity and good colloidal stability. The investigations for the synthesis of biocompatible MOF-based nanoparticles with good stability is still insufficient.
2) Furthermore, researchers must optimize the performance of MOF-DDS prior to clinical application by conducting systematic *in vivo* studies on their stability, degradation mechanics, and side effects on normal organs.

## 6 Conclusion

The current attempt was made to develop a drug carrier based on DSB-loaded MOF/Mg (DSB@MOF (Mg)) for the treatment of osteoporotic pain, bone mass loss and inflammatory response. Thus, the MOF (Mg) was constructed firstly in accordance with sonochemical approach. The prepared Mg-based MOF had admirable chemical and physical stability. A post-synthetic modification approach was employed to construct DSB@MOF (Mg) and DSB was loaded at a high rate (>25% w/w). According to drug and ion release tests, DSB@MOF (Mg) had satisfactory controlled release of Mg and DSB in solution. The results of MTT assay exhibited no cytotoxicity for the new DDS. Therefore, DSB@MOF (Mg) can be promising as a suitable candidate for relieving bone pain caused by osteoporosis, with anti-inflammatory and ossification-reinforcing functions.

## Data Availability

The original contributions presented in the study are included in the article/supplementary material, further inquiries can be directed to the corresponding author.
